# Open versus arthroscopic release for lateral patellar compression syndrome: a randomized-controlled trial

**DOI:** 10.1007/s00402-021-03878-0

**Published:** 2021-04-07

**Authors:** Sherwan A. Hamawandi, Hazhar I. Amin, Ameer Kadhim Al-Humairi

**Affiliations:** 1 FIBMS Orthopedic Surgery, Head of Orthopaedic Department, College of Medicine, Hawler Medical University, Erbil, Iraq; 2 Orthopedic Department, Erbil Teaching Hospital, Erbil, Iraq; 3 Department of Community Medicine, College of Medicine, University of Babylon, Hilla, Iraq

**Keywords:** Lateral patellar compression syndrome, Arthroscopic release, Open release, Lateral patellar retinaculum, Lysholm knee scoring scale

## Abstract

**Introduction:**

Lateral patellar compression syndrome is one of the causes of anterior knee pain in young adults and resulted from tight lateral patellar retinaculum. The aim of our study is to compare between open and arthroscopic release of lateral patellar compression syndrome in relation of functional outcome, time of surgical procedure, length of hospital stays, intraoperative and postoperative complications as bleeding, infection, recurrence, and patellar instability with 2 years of follow-up.

**Materials and methods:**

80 patients, age (21–49 years), were divided randomly into 2 groups (A and B). Group A (40 patients) were treated with open release. Group B (40 patients) were treated by arthroscopic release. All these patients are diagnosed as lateral patellar compression syndrome depending on clinical features and MRI. All patients were assessed by Lysholm knee scoring scale before surgery and at periods of 2, 6 weeks, 6, 12, and 24 months after surgery.

**Results:**

There is significant difference in functional outcome, measured by Lysholm knee scoring scale, between preoperative and postoperative assessment periods in both groups (*P* < 0.001). There is significantly better functional outcome at 2 years of follow-up with arthroscopic release (*P* = 0.018). There is no recurrence in both groups, but there were 4 patients develop medial patellar instability in the group of open release.

**Conclusion:**

Both open and arthroscopic lateral release for patients with isolated lateral patellar compression syndrome can be effective surgical procedures, but arthroscopic release can achieve better functional outcome.

*Trial registration:* NCT, NCT04130412. Retrospectively registered on 3rd of June, 2020 at *ClinicalTrials.gov.*

## Introduction

Lateral patellar compression syndrome is one of the causes of anterior knee pain which is one of the common orthopedic problems in young adults. In lateral patellar compression syndrome, the tight lateral patellar retinaculum results in overload of lateral side of the patellofemoral joint that leads to pain with consequent degeneration [1–6].

When the conservative treatment of lateral patellar compression syndrome failed, surgical treatment can be done by release of the tight lateral patellar retinaculum either by open or arthroscopic technique. The lateral patellar retinacular release can be done by either outside-in technique through open procedure or inside-out technique arthroscopically. Open technique can be done by release of lateral patellar retinaculum through mini longitudinal lateral incision or lateral lengthening with a variety of procedures like using of rotational flap of iliotibial band or suturing of the superficial layer with the deep layer of the retinaculum. Arthroscopic technique is done by release of lateral patellar retinaculum from the synovial side to the subcutaneous side [7–13].

When we reviewed previous literatures on the lateral patellar release for lateral patellar compression syndrome, we found variable results with many postoperative complications like recurrence of lateral compression syndrome and medial patellar instability [1, 12–23].

Many studies compared arthroscopic lateral release with lateral lengthening for treatment of lateral patellar compression syndrome but limited studies compared open retinacular release with arthroscopic release for lateral patellar compression syndrome [24–26].

The aim of this study is to compare the effectiveness of arthroscopic lateral patellar release with open release for lateral patellar compression syndrome regarding the functional outcome, time of surgical procedure, length of hospital staying, intraoperative and postoperative complications of bleeding, infection, recurrence, and patellar instability.

## Materials and methods

### Inclusion criteria

Inclusion criteria involve patients presented with lateral patellar compression syndrome that is proved by the following criteria and failed to conservative treatment of quadriceps strengthening exercise and non-steroidal anti-inflammatory analgesics for 6 months [12, 23].

#### Clinical features

1. Maximal pain and tenderness over the lateral margin of the patella [24, 27].
2. Abnormal patellar tilt test: when the patella cannot be lifted from the lateral femoral condyle with extended knee by the examiner [24, 27].
3. Abnormal medial patellar glide test: when the patella cannot be shifted by one or more quadrants medially by the examiner with knee flexion 10 degrees) [24, 27].

#### MRI features

1. Patellar translation relative to the femur usually occurs more laterally than medially. Subluxation/translation is measured as the distance between perpendicular lines drawn on an axial image; one from the medial edge of the patella and another one through the most anterior part of the medial femoral condyle. A 2 mm distance is the upper accepted limit of normal [28].
2. Abnormal patellar tilt, which may present with or without patellar translation, is the most closely related radiologically to lateral patellar compression syndrome. The patellofemoral angle is measured at the level of the patellar midpoint using the same method that was used on plain radiograph on sagittal imaging. It should measure more than 8° and opens laterally, if less than 8° or opens medially; it is considered abnormal [28].

#### Arthroscopy

Before doing lateral release, all patients in both groups were assessed by arthroscopy to see how the patella touching the lateral femoral condyle more than medial femoral condyle with knee movement in flexion and extension as well as exclude other pathologies.

### Exclusion criteria

Exclusion criteria include:

1. Smoking
2. Patellar instability: patient has medial or lateral glide test of 3 or more quadrants or history of patellar dislocation [24].
3. Diabetes mellitus
4. Ligament hyperlaxity based on Beighton’s criteria [29].
5. Pathological femoral anteversion or tibial torsion by Staheli’test [24, 30].
6. Q-angle more than 20 degrees [24, 31].
7. Knee osteoarthritis or patellofemoral osteoarthritis more than stage I [24].
8. Previous knee surgery or infection.
9. Outerbridge Grade 3 and 4 chondropathy.
10. Patella alta or trochlear dysplasia [24].

### Patients

Eighty patients, age 21–49 years, were involved in this study and were diagnosed to have lateral patellar compression syndrome depending on clinical features, MRI, and diagnostic arthroscopy. All patient had failed conservative measures of quadriceps strengthening exercise and analgesics for 6 months. Patients were divided into two groups randomly by entering the names of the patients into an excel file and by computer system, the patients were arranged randomly in a list, then patients with odd number sequences were regarded as group A, and patients with even number sequences were regarded as group B. Group A (40 patients) were treated with open release after diagnostic arthroscopy and Group B (40 patients) were treated by arthroscopic release. Randomization ensured even distribution of patients on two groups at 1:1 allocation. The surgeon was blind to which group was the patient belonged as the sealed envelope which contained the patient’s group was opened by the surgeon at time of patient entered to the operative room.

### Intervention

Under general or spinal anesthesia, patient was in supine position. Pneumatic tourniquet was applied on the upper thigh with leg holder. Through anterolateral and anteromedial portals, diagnostic arthroscopy was done in all patients (both groups A and B) and checking of all compartments of the knee was done. The patellotrochlear engagement was assessed especially at 30–40 degrees of knee flexion.

In group A, open release of the lateral patellar retinaculum was done through about 3 cm incision on the lateral side of the patella starting from the level of the lateral border of the middle of the patella about 2 cm laterally and extending distally to the level of lower border of the patella and the lateral retinaculum was cut longitudinally about 2 cm length and the wound was closed with drain after deflating the tourniquet and securing the hemostasis.

In group B, release of the tight lateral retinaculum was done arthroscopically by using a hook knife and electrocautery with continuous monitoring of the patellotrochlear movement during knee flexion and extension to avoid excessive release.

### Postoperative care

Knee exercise started as soon as pain was tolerated in the same day with gradual weight bearing as tolerated. Most of the patients were discharged home in the same day in group B while in the next day in group A after removal of the drain. Wound stitches were removed after 14 days postoperatively.

### Comparison

We compared group A and group B regarding primary outcome measure and secondary outcome measures.

### Outcome measures

#### Primary outcome measure, Lysholm knee score

We assessed all patients in both groups A and B for functional outcome measured by Lysholm knee score preoperatively and postoperatively at periods of 2, 6 weeks, 6, 12, and 24 months.

#### Secondary outcome measures

Involved duration of operation, length of hospital stays, intraoperative complications and postoperative complications of bleeding and infection, recurrence, and medial patellar instability.

### Study design

This study is single blind, randomized control trial. It was carried in a tertiary orthopedic hospital. The study was conducted between March, 2016 and October, 2017 with 2 years of follow-up until November, 2019.

### Lysholm Knee Scoring Scale

It is patient-reported instrument that consists of subscales for pain, instability, locking, swelling, limp, stair climbing, squatting, and need for support. Eight sections are assessed to produce an overall score on a scale of 0–100. Then, an assignment is given as “excellent” for 95 to 100 points; “good” for 85 to 94 points, “fair” for 65 to 84 points, or “poor” for less than 65 points [32].

### Statistical data analysis

Statistical analysis was carried out using SPSS version 21. Categorical variables were presented as frequencies and percentages. Continuous variables were presented as (Means ± SD). Student t test was used to compare means between two groups. Paired t test was used to compare means for paired reading. Pearson’s chi-square (χ^2^) was used to find the association between categorical variables. A *p* value of ≤ 0.05 was considered as significant.

## Results

### Demographic data

This study involved 80 patients; 39 males (48.7%) and 41 females (51.3%) with age (21–49) years and the mean of age was (39.8 ± 6.57).

They were divided randomly into 2 groups; Group A: 40 patients with equal male and female ratio and mean age was (39.9). Group B: 19 males and 21 females and mean age was (39.7).

There was no significant difference between two groups regarding gender (*P* = 0.823) and age (*P* = 893).

### Primary outcome measure: (Lysholm knee scoring scale)

When we compared preoperative mean of Lysholm knee scoring scale (58.22) with means of LKSS at 5 postoperative assessment periods; (83.88, 85.85, 86.53, 86.90, and 86.95 at 2, 6 weeks, 6, 12, and 24 months, respectively) in group A, we found that there were significant differences between the preoperative assessment and 5 postoperative assessment periods (*P* < 0.001), as shown in Table 1.

**Table 1:** The mean differences of (LKSS) between preoperative and postoperative assessments in five time periods for group A and B

Study variables	Periods of assessment	*N*	Mean	SD	Paired *t* test	*P* value
LKSS Group A	LKSS preoperatively	40	58.22	3.17	− 16.35	** < 0.001٭**
	LKSS 2 weeks postoperatively	40	83.88	10.62		
	LKSS preoperatively	40	58.22	3.17	− 18.50	** < 0.001٭**
	LKSS 6 weeks postoperatively	40	85.85	10.31		
	LKSS preoperatively	40	58.22	3.17	− 19.60	** < 0.001٭**
	LKSS 6 months postoperatively	40	86.53	10.00		
	LKSS preoperatively	40	58.22	3.17	− 20.32	** < 0.001٭**
	LKSS 1 year postoperatively	40	86.90	9.84		
	LKSS preoperatively	40	58.22	3.17	− 20.45	** < 0.001٭**
	LKSS 2 years postoperatively	40	86.95	9.79		
LKSS Group B	LKSS preoperatively	40	58.55	3.12	− 26.89	** < 0.001٭**
	LKSS 2 weeks postoperatively	40	88.13	5.75		
	LKSS preoperatively	40	58.55	3.12	− 29.23	** < 0.001٭**
	LKSS 6 weeks postoperatively	40	89.68	5.53		
	LKSS preoperatively	40	58.55	3.12	− 30.16	** < 0.001٭**
	LKSS 6 months postoperatively	40	89.88	5.41		
	LKSS preoperatively	40	58.55	3.12	− 31.38	** < 0.001٭**
	LKSS 1 year postoperatively	40	90.32	5.26		
	LKSS preoperatively	40	58.55	3.12	− 31.59	** < 0.001٭**
	LKSS 2 years postoperatively	40	90.35	5.22		

When we compared pre-operative mean of Lysholm knee scoring scale (58.55) with mean of LKSS at 5 postoperative assessment periods; (88.13, 89.68, 89.88, 90.32, and 90.35 at 2, 6 weeks, 6, 12, and 24 months, respectively) in group B, we found that there were significant differences between the preoperative assessment and 5 postoperative assessment periods (*P* < 0.001), as shown in Table 1

**Fig. 1:**
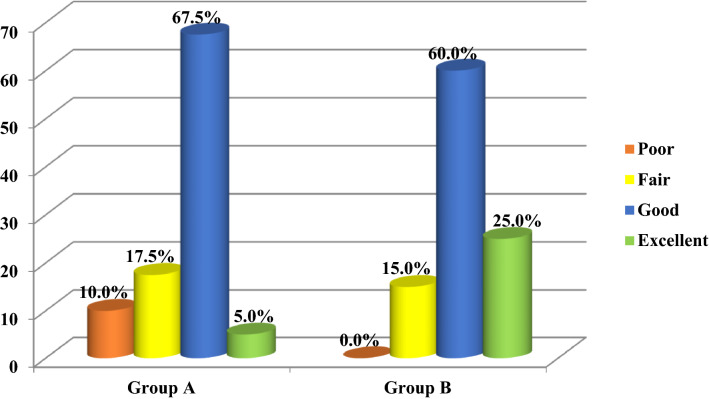
The association between LKSS in 2 years postoperatively and study groups

There was significant association between LKSS in 2 years of follow-up postoperatively and study groups **(P = 0.018٭)** as shown in Fig. 1

Symbol * means statistically significant

### Secondary outcome measures

#### Time of surgical procedure and length of hospital stay after operation

We found that the mean of the duration of surgery (in minutes) in group A was (43.12), while it was (30.50) in group B and this difference is statistically significant (*P* < 0.001), as shown in Table 2.

**Table 2:** The mean differences of duration of operation and length of hospital stay between study groups

Study variables	Study groups	*N*	Mean	SD	*t* test	*P* value	95% CI
Duration of operation (minutes)	Group A	40	43.12	2.45	18.52	** < 0.001٭**	**11.26–13.98**
	Group B	40	30.50	3.54			
Length of stay (days)	Group A	40	2.17	0.38	13.86	** < 0.001٭**	**0.92–1.22**
	Group B	40	1.10	0.30			

Symbol * means statistically significant

The mean of the length of hospital stay (in days) was (2.17), while it was (1.1) in group B and the difference is statistically significant (*P* < 0.001), as shown in Table 2.

#### Intraoperative and postoperative complications

We have 5 patients in group A got intraoperative complication of opening of the joint capsule during separation of the lateral retinaculum and it was sutured in all these patients without any sequels. There was no patient got injury of the skin overlying the released retinaculum in group B. There was no significant difference in intraoperative complication between group A and group B (*P* = 0.055), as shown in Table 3.

**Table 3:** The association between intraoperative and postoperative complications and study group

Intraoperative and postoperative complications	Study group		*P* value
	Group A	Group B	
Intraoperative complications			
Present	5 (12.5)	0 (0.0)	0.055
Absent	35 (87.5)	40 (100.0)	
Total	40 (100.0)	40 (100.0)	
Superficial wound infection			
Present	1 (2.5)	0 (0.0)	1.000
Absent	39 (97.5)	40 (100.0)	
Total	40 (100.0)	40 (100.0)	
Hemarthrosis			
Present	0 (0.0)	2 (5.0)	0.494
Absent	40 (100.0)	38 (95.0)	
Total	40 (100.0)	40 (100.0)	
Medial patellar instability			
Present	4 (10.0)	0 (0.0)	0.116
Absent	36 (90.0)	40 (100.0)	
Total	40 (100.0)	40 (100.0)	

*p value ≤ 0.05 was significant

One patient in group A got superficial wound infection which was treated by daily dressing and oral antibiotic. There was no significant difference in postoperative complication of superficial wound infection between two groups (*P* = 1.000), as shown in Table 3.

Two patients in group B got postoperative hemarthrosis and was treated by aspiration and firm bandage. There was no significant difference in postoperative complication of hemarthrosis between two groups (*P* = 0.494) as shown in Table 3.

Four patients in group A got medial patellar instability; which was diagnosed by:

1. Medial patellar translation of three or more quadrants of patellar width on Medial Patellar Glide test.
2. Positive Gravity Subluxation test by asking the patient to lie down on lateral position with the affected limb up and extended, and then, the examiner pushes on the patella to displace it medially and outside the trochlear groove while asking the patient to keep the patella in its position by contracting the quadriceps, if the patient cannot keep the patella in its position in the trochlear groove, so the patient has medial patellar instability.

Medial patellar instability is defined as medial patellar translation of three or more quadrants of patellar width on Medial Patellar Glide test with positive Gravity Subluxation test according to Nonweiler and DeLee [16, 24]. There were four patients developed medial patellar instability in group A, while there was no patient developed medial patellar instability in group B. Statistically, there was no significant difference between two groups regarding medial patellar instability, as shown in Table 3 (*P* = 0.116), but we think that it was of interest that four patients developed medial patellar instability.

Recurrence is defined as painful Passive Patellar Tilt test with Medial Patellar Glide test of less than 1 quadrant of patellar width according to Kolowich et al. [24, 33].

We check all patients in both groups for any recurrence of lateral patellar compression syndrome during 2 year follow-up using Passive Patellar Tilt test with Medial Patellar Glide test, and fortunately, we have no recurrence in both groups.

## Discussion

In current study, excellent and good results were achieved in 85% in arthroscopic release and 72.5% in open release, while Lattermann et al. [25] study showed that good result was achieved in 76%. There was significant difference between open and arthroscopic release regarding postoperative knee pain and functional outcome in the current study, while Lattermann et al. showed no significant differences between open and arthroscopic release in their systematic review of previous studies and they recommended the need for randomized-controlled trial and this what we planned to achieve in current study.

Pagenstert et al. [24] studied 14 patients who underwent open lateral release for lateral patellar compression syndrome with follow-up for 2 years, and they found recurrence in two cases and medial patellar instability in 5 cases, while in the current study, we had no recurrence but medial patellar instability occurred in four cases with same follow-up period of Pagenstert et al.’s study.

Sahu et al. [34] studied 33 patients who underwent open lateral release for lateral patellar compression syndrome and they found that satisfactory result was achieved in over 75% with no postoperative complication of hemarthrosis, while in the current study, we have satisfactory result in 72.5% with no postoperative complication in open release patients. Sahu et al. emphasized in their study that arthroscopy should always be done to exclude severe arthritic changes to avoid poor results and this what we did in our study, so all patients were assessed clinically, radiographically, and arthroscopically for arthritic changes and those more than grade I were excluded for the study.

In the current study, we had poor results in 10% of patients in group A, while no poor results were found in group B as shown in Fig. 1, and this was caused by the complication of medial patellar instability which represents the most interesting point making arthroscopic lateral release superior to open release for lateral patellar compression syndrome.

## Limitations

1/We did not do sample size calculation, because we have no exact incidence of lateral patellar compression syndrome and we asked other centers to refer those patients with this syndrome to our center for making this randomized-controlled trial.

2/We recommend larger sample size with long-term follow-up.

## Conclusion

Both open and arthroscopic lateral release for patients with lateral patellar compression syndrome (without instability, limb malalignment, or dysplasia) are effective surgical procedures, but arthroscopic release can achieve better functional outcome than open release with less risk of development of postoperative patellar instability.

## Data Availability

The datasets used and analyzed during the current study are available from the corresponding author on reasonable request.

## Abbreviations

MRI: Magnetic resonance imaging
LKSS: Lysholm knee scoring scale
Q- angle: Quadriceps angle
